# Fellow-Workers and the Roll of Honour

**Published:** 1915-11-06

**Authors:** 


### ROUND THE WORLD.

[The Editor invites Early Information and Contributions Marked "Fellow-Workers."]

### Fellow-Workers and the Roll of Honour.

The lists issued on October 29 of various decorations, appointments, and promotions for distinguished service in the field include the names of several officers of the Indian Medical Service and of the R.A.M.C. Thus Colonel Patrick Hehir, M.D., F.R.C.S., of the Indian Medical Service, and Lieutenant-Colonel John Hennessy, M.B., R.A.M.C., are appointed Companions of the Order of the Bath. Lieutenant-Colonel Walter George Pridmore, M.B., Indian Medical Service, has been appointed a Companion of the Order of St. Michael and St. George.

Captain Bertram Sibbald Finn, of the New Zealand Medical Corps, has been appointed a Companion of the Distinguished Service Order "for conspicuous devotion to duty in the Gallipoli Peninsula during the operations from August 6 to 9, 1915, when he worked day and night with unceasing zeal and without rest evacuating the wounded. His work was carried out under continuous fire, on one occasion the dressing station being heavily shelled for an hour, and many assistants and wounded being hit. Owing to Captain Finn's efforts the wounded lying in the more exposed positions were got into a place of greater safety." Truly a noble record.

Temporary Lieutenant Allan Noel Minns, of the 39th Field Ambulance, Royal Army Medical Corps, has gained his D.S.O. under the following circumstances described in the official lists of October 29: "For conspicuous gallantry and devotion to duty at Suvla Bay, Gallipoli Peninsula, on August 30, 1915, when attending to the wounded under heavy shrapnel fire. Another officer who was assisting him was killed. Lieutenant Minns later returned to the dressing station, took out twelve stretcher squads, and brought in twenty-four wounded men." Lieutenant Minns, whose home is at Thetford, became an L.M.S.S.A. Lond. in 1914.

The leaders of the medical profession seem to have given many of their sons in their country's cause. Sir Lauder Brunton's youngest son, Lieutenant Edward Henry Pollock Brunton, R.A.M.C., has fallen in France. A student of Trinity College, Dublin, and St. Bartholomew's Hospital—where his distinguished father carried out much of the work which has made him famous—Lieutenant Brunton qualified in 1913, and then became an M.B., B.C. Cantab. Before joining the Army he had been house physician at "Bart.'s" and house surgeon at the Royal Portsmouth Hospital.

Lieutenant Robert Montgomery, R.A.M.C., another of the medical officers to fall in the move forward on the Continent, joined the Special Reserve of the R.A.M.C. after qualifying M.B., B.Ch. at Edinburgh University, and was mobilised in the early days of the war. His experiences before this included a spell of private practice, and work as medical officer to the troops stationed at Edinburgh Castle.

Captain George Leonard Grant, R.A.M.C., recently killed, had had a somewhat varied military experience. Although a duly qualified surgeon, he went abroad in the ranks of a combatant unit, but was soon transferred to work for which he was particularly fitted, and given a commission in the R.A.M.C. The son of a medical practitioner—Dr. Grant, of New Southgate—Captain Grant was educated successively at Epsom, Cambridge, and the London Hospital.

St. Mary's Hospital has lost an assistant pathologist by the death of Lieutenant Edward Jocelyn Nangle, R.A.M.C., who left the work of that post to take a temporary commission as a medical officer when war broke out. A South African, he commenced his scientific studies at Cambridge, subsequently proceeding to St. Mary's for his final work.

Captain Gurney White Buxton, R.A.M.C. (T.), was in practice at Fenny Stratford when the call came for him to serve the cause for which he has just given his life. He had been a Territorial officer for some years, and attached to the 2nd South Midland Mounted Brigade Field Ambulance. Captain Gurney was an old "Bart.'s" man, and qualified in 1891.

Captain Thomas Henry Stanley Bell, R.A.M.C., lately killed, qualified by taking the M.B., B.Ch. at Edinburgh only last year. He had had previous experience of military surgery through service with the Serbian Army during the Balkan War, and on the outbreak of hostilities in the summer of 1914 he took a commission in the Special Reserve (R.A.M.C.).

Captain Sydney Francis Macalpine Cesari, R.A.M.C., has also been killed in recent fighting. He became an M.B., B.Ch. of Edinburgh three years ago, and subsequently held resident posts at the Edinburgh Royal Infirmary and at Greenock Infirmary.

Captain John Harry Meers, R.A.M.C., who has died of wounds, was appointed temporary captain in the Royal Army Medical Corps in August last, and was attached to the 1st Battalion of the Loyal North Lancashire Regiment. An old St. Mary's man, he became an M.R.C.S., L.R.C.P. Lond. five years ago. For a period he was assistant medical officer at the Islington Infirmary.

Lieutenant Bertram Chiene Letts, R.A.M.C., who died at Alexandria on October 21 from dysentery contracted in the Dardanelles, was the only child of Professor E. A. Letts, who has occupied the Chemistry Chair in the Queen's College, Belfast, and in the Queen's University since 1879. Lieutenant Letts became an M.B., B.Ch., B.A.O. Belfast in 1913.

Captain Victor Cleeve Martyn, R.A.M.C., whose name appeared in the list of wounded on November 2, became an M.R.C.S., L.R.C.P. Lond. in 1911. For a time he was a clinical assistant at the Central London Throat and Ear Hospital and a house surgeon at the London Temperance Hospital. His publications include articles on the treatment of diphtheria.

Sir Anthony Bowlby, C.M.G., F.R.C.S. Eng., who went abroad in the latter part of last year as consulting surgeon to the Expeditionary Force with the rank of colonel, has lately been promoted to be a Surgeon-General.

